# Effect of caffeine on sleep and behaviour in nursing home residents with dementia

**DOI:** 10.1007/s41999-018-0115-6

**Published:** 2018-10-01

**Authors:** L. M. M. de Pooter-Stijnman, S. Vrijkotte, M. Smalbrugge

**Affiliations:** 1 Department of General Practice and Elderly Care Medicine, VUmc/Amsterdam Public Health Research Institute, Amsterdam, The Netherlands; 2 Stichting Zorggroep Solis, Deventer, The Netherlands; 3 Department Human Physiology Research Group, Vrije Universiteit Brussel, Brussels, Belgium; 4 Vital Signs and Performance Monitoring (VIPER), Royal Military Academy, Brussels, Belgium

**Keywords:** Dementia, Caffeine, Sleep, Behavioural symptoms

## Abstract

**Background and goal:**

Sleep problems and challenging behavioural symptoms are frequently reported among nursing home residents with dementia. Coffee with caffeine is consumed frequently by these residents and can have a negative effect on sleep and behaviour in older persons with dementia. In this interventional study, the effect of caffeine reduction on sleep and challenging behavioural symptoms in nursing home residents with dementia was investigated.

**Methods:**

In 21 nursing home residents with dementia living in 1 dementia special care unit, caffeine was gradually eliminated in the afternoon and evening. During pre-intervention and post-intervention period the care workers daily scored sleep by a specially developed sleep questionnaire. Behavioural symptoms were scored in the afternoon and evening using four items of the NPI-NH: agitation/aggression, apathy, irritability and aberrant motor behaviour.

**Results:**

A significant improvement in sleep scores (Wilcoxon signed-rank test, *p* = 0.015) and apathy (Wilcoxon signed-rank test, *p* = 0.020) was found after eliminating caffeine intake in the afternoon and evening. No significant changes occurred in agitation/aggression, irritability and aberrant motor behaviour.

**Discussion:**

This pre-post pilot study found a significant positive effect of caffeine reduction on sleep and apathy and warrants further investigation in a larger controlled study.

## Introduction

Sleep problems occur in 25–35% of patients with Alzheimer dementia [1]. Sleep disorders are associated with cognitive decline, behavioural symptoms, reduction of quality of life and they place an extra burden on informal caregivers and nursing staff [1, 2]. The etiology of sleep problems in dementia is multifactorial. Decrease in acetylcholine is assumed to be one of the factors [1]. Caffeine gives a prolonged sleep latency (i.e. time to fall asleep) and attenuates the homeostatic regulation of sleep by adenosine receptor antagonism [3]. Caffeine gives a reduction of electroencephalographic slow-wave activity (i.e. deep sleep) [3, 4]. For the REM-sleep, both a decrease and an increase are reported [5, 6].

Challenging behavioural symptoms also frequently occur in patients with dementia in a nursing home, with 81% of these patients having one or more clinically relevant behavioural symptoms [7]. Agitation/aggression, apathy, irritability and aberrant motor behaviour are the most observed behavioural symptoms with a prevalence of around 30% [7]. Sundowning is the phenomenon where behavioural symptoms in people with dementia increase in severity at the end of the afternoon and early evening and is common among nursing home residents with dementia [8, 9].

Coffee with caffeine is widely consumed even in nursing homes and caffeine is a stimulant that can have a varying and individual effect through its influence on different neurotransmitters [10]. In healthy adults, caffeine improves concentration and reduces fatigue [11]. But in excessive amounts or in vulnerable groups, namely people with psychiatric disorders, it can increase or induce anxiety and insomnia [11]. In general, healthy adults adjust their caffeine consumption themselves, but persons with dementia who depend on others are less able to do this themselves and they are therefore more likely to experience negative effects [12]. Kromhout describes in an observational pilot study in people with dementia in the nursing home a positive correlation between caffeine and getting up at night and a negative correlation between caffeine and apathy and aberrant motor behaviour [10]. A single-subject trial suggests that caffeine affects behavioural symptoms in residents with dementia, but that this may be very individual [12]. Non-pharmacological interventions for sleep problems in persons with dementia like reducing caffeine intake can be useful, because in this group sedatives are associated with various health risks and a recent randomized controlled trial (RCT) showed that melatonin was not effective [13].

In summary, caffeine can have a negative effect on sleep and behaviour in older people, especially older persons with dementia. But the effect of reducing caffeine is not known, because no interventional study has been previously carried out in persons with dementia in the nursing home. Several hypotheses can be formulated on the expected effects of caffeine reduction. Due to its stimulating effects, caffeine reduction might improve sleep and might change behavioural symptoms. In this pilot study with pre- and post-comparison, we investigate whether eliminating caffeine intake in the afternoon and evening has an effect on sleep and behavioural symptoms of nursing home residents with dementia.

## Methods

### Study design and study population

A pilot intervention study with pre- and post-comparison was conducted at a dementia special care unit of a Dutch nursing home with 28 residents. The local ethics committee Universitair Netwerk Ouderenzorg–Commissie Solis (Uno-CoSo) gave permission for the study. Written informed consent was obtained through the representatives of the participants. Two participants were also able to give oral consent themselves. The others were not able to give consent themselves. The participants and representatives were free to discontinue their participation without giving a reason. Inclusion criteria were the diagnosis of dementia and the use of at least one cup of coffee per day. Exclusion criteria were no informed consent, life expectancy  <  1 month or living < 4 weeks in the unit at the start of the study.

### Intervention

The study lasted 6 weeks and consisted of three phases of 2 weeks each: baseline (pre-intervention), wash-out period of caffeine (introduction of intervention) and new situation (post-intervention introduction). In the pre-intervention phase, nothing changed in terms of coffee consumption. During the wash-out period, caffeine intake was gradually reduced to the new situation. In the post-intervention phase participants took caffeinated coffee in the morning (from 6 a.m. to 12 a.m) and decaffeinated coffee from 12 a.m.

We chose to maintain caffeinated coffee in the morning, because of possible beneficial effects in the morning and because negative effects on behaviour symptoms and sleep were supposed to be more likely in the afternoon, evening and night. During the study, participants drank instant coffee from a coffee vending machine in a standard cup of 150 ml. The caffeinated coffee contained about 75 mg of caffeine per cup [14].

#### Blinding

All the staff, including care workers and catering staff, were informed which residents participated in the study. They also knew that the effect of caffeine on sleep and behaviour was investigated, but they were not aware of the design of the study and the wash-out schedule. The participants received coffee from the same coffee machine, which contained caffeinated one time and decaffeinated coffee the other time. The care workers were unaware which type of coffee was in the coffee machine. Independent persons switched the coffee packages and made sure that the machine would not be empty unplanned. We made an exchange schedule with real and fake switches. The coffee packages were exchanged at the real switches. The packages were not changed at the fake switches, but the independent persons were at the unit and opened the coffee machine. The fake switches were performed to keep the care workers unaware of the type of coffee. The residents who did not participate in the study were given caffeinated coffee from another unit.

### Variables and measurements

#### Independent variables

##### Baseline characteristics

Baseline characteristics: sex, age, type of dementia, duration of dementia, renal function, medical history of anxiety disorder, depression or delirium and use of psychotropic medication were obtained from the medical records. The severity of dementia was scored with the Reisberg Global Deterioration Scale [15] by the responsible care worker and the author. Residents have a free choice for their beverages in the nursing home, but from the information provided by the care workers, the consumption of coffee and tea in general hardly differed per participant per day. Before the start of the study, the head of unit informed us about the number of cups of coffee and tea per day per participant.

##### Confounders

Possible confounders, namely incidence of physical illness, delirium, psychosocial events, change in tea consumption or change in fixed use of psychotropic medication during the study and in the 2 weeks prior of the start of the study were noted.

#### Outcome variables (dependent variables)

##### Sleep

We used a specially developed questionnaire for sleep which was scored at the night rounds that the care workers already conducted (11:00 p.m., 1:00 a.m., 3:00 a.m., and 5:00 a.m.). The questionnaire was filled in every night.

The on-duty care worker scored on a four-point scale for each round whether the participant was quietly sleeping (score 1), restlessly sleeping (score 2), quietly awake (score 3) or restlessly awake (score 4). An example of restless sleep is movements of arms and legs. Quietly awake, for example, is quietly lying awake in bed. Examples of restlessly awake are being awake with emotionality, anxiety, calling or wandering around. The care workers were also asked to note the influence on sleep due to behaviour of other residents and details between the rounds that alarmed the care workers such as wandering or calling. The care workers were instructed in scoring the questionnaire.

Based on the sleep scores, two new variables were defined: having a good night sleep or having a poor night sleep. A poor night sleep was defined as ≥ 2 sleep score of 4 or 1 × sleep score of 3 and 1 × sleep score of 4 or ≥ 3 × sleep score of ≥ 3 or 4 × sleep score of 2. The definitions were made on general assumptions about a good and a poor night sleep.

##### Behavioural symptoms

Behavioural symptoms were scored using an adaptation of the Neuro Psychiatric Inventory-Nursing Home edition (NPI-NH) [16, 17]. The NPI-NH is a valid observation scale for neuropsychiatric symptoms in people with dementia in the nursing home and consists of 12 behavioural domains [18, 19]. Based on the expected effect of caffeine and the previously found associations by Kromhout in 2014, we chose to score four domains of the NPI-NH: agitation/aggression, apathy, irritability and aberrant motor behaviour [10].

The NPI-NH consists per domain of a gate question about the presence or absence of the symptom of that domain (domain score) and a score for frequency, severity and emotional burden for the care worker. A total score per domain can be calculated with the frequency and severity [16, 17]. The NPI-NH is normally based on a period of multiple subsequent weeks; however, we daily scored the NPI-NH between 3:00 p.m. and 11:00 p.m., taking into account the time when sundowning occurs [8]. This means that frequency and total score cannot be measured, but only the domain score, severity and emotional burden could be scored with regard to the mentioned time period. The care workers were given instructions on how to score the NPI-NH.

##### Psychotropic medication

During the course of the study, we collected the administration lists of the medication to compare the as-needed use of antipsychotics, anxiolytics and hypnotics (ATC codes) in the pre- and post-intervention period.

### Statistical analysis

The prevalence of sleep problems and behavioural symptoms was described using descriptive statistics. Differences between pre- and post-intervention period were compared with SPSS 25.0 using a significance level of 0.05.

The differences in NPI-NH scores between pre-and post-intervention period were compared using the non-parametrically paired Wilcoxon signed-rank test. Three analyses for each of the four domains were performed: for domain score, severity and emotional burden.

The differences in sleep score between pre- and post-intervention period were tested for a statistically significant difference with the non-parametric paired Wilcoxon signed-rank test.

With the as-needed use of psychotropic medication per ATC-code we compared the average number of times the medication was given in the pre- and post-intervention period using the paired *t* test.

## Results

### Participants

Based on the inclusion and exclusion criteria, seven residents were excluded because of no coffee use (*n* = 2), no informed consent (*n* = 2), no diagnosis of dementia (*n* = 1) or living < 4 weeks in the unit (*n* = 2). The remaining 21 residents participated in the study. The baseline characteristics of the study population are described in Table 1. Nine participants did not use psychotropic medication. Although the remaining 12 persons used one of more types of psychotropic medication, no changes occurred during the study. In the week before the start of the study, melatonin was stopped in one person due to drowsiness. One participant died during the study. No post-measurement was taken yet, which is why this participant was not included in the analysis.

**Table 1:** Baseline characteristics of the study population (*n* = 21)

Sex (*n*)	
Male	4
Female	17
Age in years: mean (range)	87 (70–98)
Dementia (*n*)	
Alzheimer’s disease [13]	5
Vascular dementia (VD)	4
Mixed dementia (AD/VD)	3
Frontotemporal dementia	1
Not otherwise specified	8
Global Deterioration Scale (*n*)	
5 = Moderate dementia	9
6 = Moderately severe dementia	10
7 = Severe dementia	2
Duration of dementia in months: mean (range)	53 (3–138)
Renal function CKD-EPI: mean ml/min (range)	65 (35–90)
Medical history (*n*)	
Anxiety disorder	2
Depression	7
Delirium	6
Psychotropic medication ATC (*n*)	
Antidepressants	8
Antipsychotics	1
Anxiolytics	6
Hypnotics (benzodiazepines and melatonin)	7
Anti-epileptics	0
Anti-dementia medication	0
Caffeine consumption	
Coffee: mean units/day (range)	4 (2–5)
Tea: mean units/day (range)	1 (0–3)
Other: ice tea/energy drink/chocolate > 100 g/week	0

### Sleep

Data about sleep scores are summarized in Table 2. The total sleep score measured with the sleep questionnaires significantly improved in the post-intervention period compared with the pre-intervention period (*p* = 0.015). The number of times that quietly sleeping was scored significantly increased.

**Table 2:** Difference in sleep between pre- and post-intervention period and between pre-intervention period and wash-out period (paired Wilcoxon signed-rank test)

	Pre (%)	Post (%)	Difference^a^	Wash-out period	Difference^b^
Total sleep score			*p* = 0.015*		*p* = 0.358
Score 1 = quiet sleep	83.1	86.6	*p* = 0.032	82.0	*p* = 0.840
Score 2 = restless sleep	1.7	1.2	*p* = 0.509	1.9	*p* = 0.832
Score 3 = quietly awake	8.7	7.5	*p* = 0.243	8.6	*p* = 0.886
Score 4 = restlessly awake	6.5	4.7	*p* = 0.101	7.6	*p* = 0.603

*Significant difference

^a^Difference between pre- and post-intervention period

^b^Difference between pre-intervention period and wash-out period

We also conducted an analysis with the recoded variables, namely having a good night sleep and having a poor night sleep. In the post-intervention period three participants had a decrease in number of good night sleep while nine participants had an increase of good night sleep and eight had equal scores (statistically significant improvement: *p* = 0.048, paired Wilcoxon signed-rank test).

### Behaviour

The most frequent behavioural symptom was agitation/aggression. The least frequent behavioural symptom was apathy. Seven participants (35%) showed apathy one or more times during the pre-intervention period. In the post-intervention period this applied to two participants (10%). The domain score for apathy in the afternoon and evening significantly decreased in the post-intervention period (Table 3). The domain scores of the other behavioural symptoms did not differ between pre- and post-intervention period.

**Table 3:** Difference in domain score pre-intervention compared to post-intervention (paired Wilcoxon signed-rank test)

	Pre (%)^a^	Number^b^	Post (%)^a^	Number^b^	Difference
Agitation/aggression	60	1–4	50	1–8	*p* = 0.261
Apathy	35	1–2	10	1	*p* = 0.020*
Irritability	50	1–3	35	1–10	*p* = 0.314
Aberrant motor behaviour	45	1–5	45	1–6	*p* = 0.877

*Significant difference

^a^ % of persons that showed one or more times the behavioural symptom (= a positive domain score in NPI) during the pre- or post-intervention period

^b^Number of times (= days) a positive domain score was scored

The emotional burden score and the severity score did not differ statistically significant for the four behavioural symptoms, although there was a trend to a statistically significant decrease for the severity score of apathy (*p* = 0.062).

### Psychotropic medication

The average number of times that as-needed psychotropic medication from the groups of antipsychotics, anxiolytics and hypnotics (benzodiazepines) were given did not differ between pre- and post-intervention period (Table 4).

**Table 4:** (As-needed) Psychotropic medication, mean number of times given per person in 14 days (paired *t* test)

	Pre	Post	Difference
Antipsychotics (N05A)	0.25	0.40	*p* = 0.33
Anxiolytics (N05B)	1.85	2.10	*p* = 0.71
Hypnotics (N05CD)	0.35	0.35	*p* = 1.00

### Possible influence of confounders

The incidence of possible confounders or change in their presence, namely physical illness, delirium, psychosocial events, tea consumption and fixed use of psychotropic medication were negligible.

The consumption of tea and fixed use of psychotropic medication did not change. Delirium and relevant psychosocial events did not occur. Physical illness occurred in three participants in the pre-intervention period, three in the intervention period and two in the post-intervention period.

## Discussion

In this pilot intervention study in nursing home residents with dementia, a significant improvement of sleep scores and apathy was found after eliminating caffeine in the afternoon and evening. No significant changes were found in agitation/aggression, irritability, aberrant motor behaviour and in the as-needed use of antipsychotics, anxiolytics and hypnotics.

To our knowledge, this is the first interventional study in which caffeine was reduced in persons with dementia. Our study confirms the positive correlation of caffeine consumption with waking up at night that was found in the observational pilot study by Kromhout, as sleep scores improved after reduced caffeine intake. The negative correlations with aberrant motor behaviour and apathy (higher caffeine consumption associated with less aberrant motor behaviour and less apathy) in the same pilot study are not supported by our study results [10].

Sleep scores improved in our study. An explanation for the improvement of sleep scores is the improvement of slow-wave activity and build-up of sleep by eliminating caffeine intake in the afternoon and evening.

Apathy occurred in the pre-intervention period in 35% of the participants, but only once or twice per person. This makes apathy in our group a minor clinically relevant symptom compared to the other symptoms measured. Partly this may be due to underreporting of this symptom: apathy being regarded as normal for this stage of dementia. Nevertheless, a significant improvement in apathy was seen after the intervention. An explanation may be that the improvement of sleep post-intervention gives a decrease in apathy during the day. By subgroup analysis, we have examined whether apathy was more common among participants with relatively poor sleep and how this related to the pre- and post-intervention period. Out of the seven participants who scored positive on apathy in the pre-intervention period, five were among the seven least good sleepers. And when they slept better in the post-intervention period, their apathy declined.

This pilot study has some limitations. The sample size is small and concerns only the residents of one department of one nursing home. The outcomes were recorded by care workers who were aware of the trial and there was no control arm. The follow-up period is relatively short and caffeine levels in the blood were not measured. We chose to conduct the study only at one department and not to measure the caffeine levels in the blood because the effects of caffeine reduction were unknown and we did not want to burden the residents and the care workers. The sleep questionnaire we developed has not been validated, but is easy to apply and is little time-consuming because the care workers can incorporate it during their already-scheduled night rounds. Other questionnaires for sleep such as the sections of sleep of the NPI-NH and Cornell are being applied on a larger scale, but are limitedly sensitive [20]. The Sleep Disorders Inventory (SDI) has been more validated, but it takes a long time to complete for the care worker [21]. The Netherlands are in the top five of coffee consuming nations [22]. In countries where coffee is not much consumed, the intervention is less relevant. The strengths of the research are the blinding of the care workers and completeness of the data collection. The intervention is easy to implement and the costs are low. In addition, there were no adverse effects or comments about the taste of the decaffeinated coffee, which makes the introduction relatively simple. There is no pre-selection on a group of persons with many behavioural symptoms or sleep problems. Although a part of the participants slept well and did not have apathy in the pre-intervention period, we still find a significant difference in sleep scores and apathy. Caffeine therefore appears to be a strong influencing factor of both.

In conclusion, there are clear indications that eliminating caffeine intake in the afternoon and evening can improve sleep and decrease apathy in nursing home residents with dementia. As the effect of eliminating caffeine intake may be very individual, decreasing caffeine intake on trial is certainly an option for people with dementia and sleep problems or apathy combined with sleep problems. A large RCT with control group performed in several nursing homes is recommended to re-examine this pilot study hypothesis and explore whether clinically relevant effects are found again. We suggest to score sleep and behaviour by independent and fully blinded persons. It can also be investigated what the effect is when caffeine is completely stopped (during the whole day). Using the intervention on a larger scale as a standard for people with sleep problems or converting a whole dementia special care unit to decaffeinated coffee should only be done if a larger intervention study also finds positive results.
